# Past, Present, and Future of Involuntary Admission in Georgia

**DOI:** 10.1192/j.eurpsy.2022.133

**Published:** 2022-09-01

**Authors:** E. Chkonia

**Affiliations:** Tbilisi State Medical University, Psychiatry, Tbilisi, Georgia

**Keywords:** Involuntary admission, mental health law, human rights, mental health legislation

## Abstract

Since gaining independence in 1991, Georgia has struggled to transform the old-Soviet mental health care structure into a humane system to meet basic human rights standards.

The current version of the mental health law was introduced in 2007, which instituted the new practice that required court decisions for involuntary hospitalization and several practical procedures.

The Public Defender’s Office (Special reports, 2019-2021) revealed gaps and contradictions within the law that lead to human rights violations and malpractices in involuntary hospitalization.

Currently, the group of Georgian experts with international support from Expertise France- French Development Agency, at the request of the Ministry, are working on the new version of the mental health law, which will be in line with international requirements and standards.

**Disclosure:**

No significant relationships.

